# Supplementary material for: Development of a multidimensional prediction model for long-term prognostic risk in patients with acute coronary syndromes after percutaneous coronary intervention: A retrospective observational cohort study
Source: PLoS One. 2025 May 7;20(5):e0318445. doi: 10.1371/journal.pone.0318445 (PMC12057874; doi:10.1371/journal.pone.0318445)
Supplement: Attachment — Submitted filename: Response to Reviewer Comments-20250310.docx [file pone.0318445.s003.docx]

PONE-D-24-46383

Development of a multidimensional prediction model for long-term prognostic risk in patients with acute coronary syndromes after percutaneous coronary intervention：a retrospective observational cohort study

Dear Hugh Cowley,

Staff Editor
PLOS ONE

We are so glad to receive your reply to our submitted paper. Sincere thanks should be given to you, associated the reviewers for thoroughly examining the original version of our manuscript and providing important criticisms and suggestions to guide our revision. We have taken all these comments into account and tried our best to revise the manuscript, in which changes we made has been highlighted in yellow highlight. The point-by-point responses to the criticisms and suggestions are presented below.

Best regards,

Pengyu Cao, MD.

Tel.: +86 186-0446-4821

E-mail: caopy1979@outlook.com

^1^Changzhou Medical Center, Nanjing Medical University, Changzhou, 213004, Jiangsu, China

^2^The Second People's Hospital of Changzhou, the Third Affiliated Hospital of Nanjing Medical University, No. 68 Gehu Middle Road, Changzhou 213164, Jiangsu, China

### Response to Reviewer Comments

**Reviewer 1**

The presented study focuses on the development of a multidimensional predictive model for assessing long-term survival in patients who have undergone percutaneous coronary angioplasty following acute coronary syndromes. This is a cohort, observational study. The study included patients treated for myocardial infarction and unstable angina. The model was developed using selected laboratory and clinical parameters as well as indicators related to patients' physical activity. It appears that the ability to engage in physical activity and record its intensity determined the characteristics of the studied population and the values of EF and EDLV. The study was conducted correctly, and the statistical analysis was performed appropriately. However, the article requires several revisions and clarifications.

**A:** Thank you for your insightful comments regarding the focus of our study. We sincerely appreciate your feedback and would like to take this opportunity to revise and clarify our research.

**Q1:** The authors must decide whether they intend to use the term fasting blood sugar or fasting plasma glucose, as both are used interchangeably throughout the article.

**A1:** Thank you for pointing out the problems in our research, and your suggestions have made our article more rigorous. The term we ultimately chose is fasting plasma glucose (FPG). By reviewing related articles, we know that FPG refers specifically to venous plasma fasting glucose, while fasting blood sugar (FBS) refers to whole blood fasting glucose. Our data comes from the patients' venous blood lab results. We have replaced the FBS in the text with FPG and highlighted it.

**Q2:** The authors must specify whether the reported values of laboratory and echocardiographic parameters were measured at admission, at discharge, or averaged.
o The EF values in AMI are surprisingly high: EF (%), (IQR) 57.00 (55.00,60.00) 58.00 (53.00,60.00).

**A2:** Thank you for your insightful comment. To clarify, except for troponin, which was measured at admission, all other laboratory and echocardiographic parameters were assessed before discharge, after the surgical procedure. This approach was taken because many AMI patients were admitted as emergency cases, and the relevant parameters were not measured at the time of admission. In our previous manuscript, we inaccurately stated that these parameters were collected after disease onset. We have now revised this to specify that they were measured after PCI and have highlighted the changes in the text.

Regarding the EF values, the reported EF measurements were obtained before discharge, which may explain the relatively high values observed. We have performed a statistical analysis and found that 22 individuals (12%) had an EF < 50% among AMI patients. Specifically, 16 patients (10%) were in the NO-MACE group, while 6 patients (19%) were in the MACE group. The detailed statistical results have been included in Table 2 for reference.

|  | AMI (n=185) | | |
| --- | --- | --- | --- |
|  | Non-MACE (n=154) | MACE (n=31) | *P* |
| EF＜50%, n (%) | 16（10.39） | 6（19.35） | 0.270 |

(Red words in **Table 2**, page 31, yellow highlight)

**Q3:** The presented study has several limitations that require description: a. The variables did not include troponin levels. b. The duration of ischemia was not considered. c. The number of affected coronary arteries was not included. d. The type of myocardial infarction was not specified, i.e., whether it was an ST-elevation myocardial infarction (STEMI) or a non-ST-elevation myocardial infarction (NSTEMI). e. The study was conducted, and consequently, the model was developed based on a relatively small population. f. The hemodynamic status of patients at hospital admission (Killip-Kimball classification) was not considered.

**A3:** Thank you for your valuable comments. We have carefully addressed each of the suggested limitations as follows:

a. We have supplemented our analysis with troponin levels measured upon admission in AMI patients. Our results show no significant difference between the MACE and NO-MACE groups (*P* = 0.588), and this has been incorporated into Table 2.

|  | AMI (n=185) | | |
| --- | --- | --- | --- |
|  | Non-MACE (n=154) | MACE (n=31) | *P* |
| Troponin(ng/ml), (IQR) | 2.03（0.36,6.28） | 1.82（0.23,6.36） | 0.257 |

(Red words in **Table 2**, page 31, yellow highlight)

b. The patients reviewed in our study were admitted from the emergency department and underwent PCI according to the Chinese guidelines; however, the exact time from the onset of symptoms to the opening of the vessel could not be obtained from the medical records at this time.

Additionally, the patients reviewed in our study were admitted from the emergency department and underwent PCI according to the Chinese guidelines; however, the exact time from the onset of symptoms to the opening of the vessel could not be obtained from the medical records at this time. This factor may influence prognosis, we acknowledge that our study did not account for the duration of myocardial ischemia, which is a limitation.

(Red words in **Discussion** section, page 20, line 17 - line 21, yellow highlight)

c. We have already analyzed the number of stents implanted in both the AMI and UA groups, with detailed results provided in Table 2 .

|  | AMI (n=185) | | |
| --- | --- | --- | --- |
|  | Non-MACE (n=154) | MACE (n=31) | *P* |
| No. of stents, n (%) |  |  | 0.257 |
| 1 | 94(61.04) | 18(58.06) |  |
| 2 | 37(24.03) | 11(35.49) |  |
| ≥3 | 23(14.93) | 2(6.45) |  |

|  | UA (n=102) | | |
| --- | --- | --- | --- |
|  | Non-MACE (n=82) | MACE (n=20) | *P* |
| No. of stents, n (%) |  |  | 0.252 |
| 1 | 41(50.00) | 14(70.00) |  |
| 2 | 23(28.05) | 4(20.00) |  |
| ≥3 | 18(21.95) | 2(10.00) |  |

(Red words in **Table 2**, page 32, yellow highlight)

d. We have reviewed the electrocardiographic data of AMI patients and found that 121 patients were diagnosed with STEMI, while 64 patients were diagnosed with NSTEMI. Our analysis showed no significant difference between the MACE and NO-MACE groups (*P* = 0.764), and this information has now been added to Table 2.

|  | AMI (n=185) | | |
| --- | --- | --- | --- |
|  | Non-MACE (n=154) | MACE (n=31) | *P* |
| ST elevation, n (%) | 100(64.94) | 21(67.74) | 0.764 |

(Red words in **Table 2**, page 31, yellow highlight)

e. We have examined the Killip-Kimball classification of AMI patients, with 161 patients classified as Killip I, 22 as Killip II, and 2 as Killip III. There was no statistically significant difference between the MACE and NO-MACE groups (*P* = 0.760). These findings have also been incorporated into Table 2.

|  | AMI (n=185) | | |
| --- | --- | --- | --- |
|  | Non-MACE (n=154) | MACE (n=31) | *P* |
| Killip, n (%) |  |  | 0.760 |
| Ⅰ | 133(86.36) | 28(90.32) |  |
| Ⅱ or Ⅲ | 21(13.63) | 3(9.67) |  |

(Red words in **Table 2**, page 31, yellow highlight)

We appreciate your insightful suggestions, which have helped us refine our study. We have now updated the Discussion section accordingly and remain open to any further recommendations.

**Q4:** It is recommended to expand the discussion section with references to other studies describing the significance of laboratory indicators in cardiovascular risk assessment, for example:

a. https://doi.org/10.1016/j.ijcard.2024.132663.

b. <https://doi.org/10.1093/eurheartj/ehae666.1571.>

**A4:** Thank you for your valuable suggestion. We appreciate the recommended references and agree that expanding the discussion with comparisons to other studies on laboratory indicators in cardiovascular risk assessment will strengthen our manuscript. Accordingly, we have revised the Discussion section to incorporate relevant findings from these and other studies, highlighting the significance of laboratory parameters in predicting cardiovascular outcomes. The newly added references provide a broader context and further support our findings.

Several simple and easily measurable biomarkers have demonstrated substantial value in the prognostic assessment of patients with acute coronary syndrome (ACS). These parameters are straightforward to calculate, cost-effective, and obtainable through routine clinical blood tests, thus offering broad applicability and significant clinical utility. By identifying high-risk patients early, they enable tailored interventions and improved clinical outcomes. The hemoglobin-to-creatinine ratio (Hb/Cr) serves as an integrated marker of anemia and renal dysfunction, both of which contribute to chronic inflammation and hemodynamic disturbances in ACS patients, thereby influencing prognosis^[60, 61]^. Lower Hb/Cr levels have been associated with increased all-cause mortality, higher rates of reinfarction, and a greater incidence of major bleeding at one year among patients with ACS^[61]^ . However, the current study did not observe a significant predictive effect of Hb/Cr on post-percutaneous coronary intervention (PCI) outcomes. This discrepancy may be attributed to variations in patient characteristics, as most individuals enrolled in this study exhibited normal hemoglobin and creatinine levels, with no notable prevalence of anemia or renal dysfunction. The triglyceride-glucose (TyG) index, a surrogate marker for insulin resistance and dysregulated glucose-lipid metabolism, is closely linked to atherosclerotic progression. Prior research has identified the TyG index as not only predictive of atherosclerosis advancement but also as an independent prognostic indicator for major adverse cardiovascular events (MACE) and all-cause mortality in ACS patients^[62, 63]^ . Despite this, our study did not find a significant association between TyG and the prognosis of ACS patients following PCI. This lack of predictive value may stem from the study cohort's characteristics, where the prevalence of diabetes mellitus, dyslipidemia, and baseline triglyceride and fasting glucose levels did not significantly differ between the NO-MACE and MACE groups.

(Red words in **Discussion** section, page 18, line 11 - page 19, line 4, yellow highlight)

[60] Demir, M.; Kahraman, F.; Sen, T.; Astarcioglu, M. A. The Relationship of the Hemoglobin to Serum Creatinine Ratio with Long-Term Mortality in Patients with Acute Coronary Syndrome: A Retrospective Study. *Medicine (Baltimore)* **2023**, *102* (41), e35636. https://doi.org/10.1097/MD.0000000000035636.

[61] Spadafora, L.; Mattia Galli, M. G.; Marco Bernardi, M. B.; Matteo Betti, M. B.; Stefano Cacciatore, S. C.; Fabrizio D’ascenzo, F. D. A.; Gaetano De Ferrari, G. D. F.; Ovidio De Filippo, O. D. F.; Giacomo Frati, G. F.; Salvatore Brugaletta, S. B.; Alberto Polimeni, A. P.; Maciej Banach, M. B.; Pierre Sabouret, P. S.; Gianmarco Sarto, G. S.; Giuseppe Biondi-Zoccai, G. B. Z. Hemoglobin/Creatinine Ratio as Long- Term Prognostic Marker in Acute Coronary Syndromes: A Praise Registry Study. *Eur. Heart J.* **2024**, *45* (Supplement_1), ehae666.1571. https://doi.org/10.1093/eurheartj/ehae666.1571.

[62] Wang, L.; Cong, H.-L.; Zhang, J.-X.; Hu, Y.-C.; Wei, A.; Zhang, Y.-Y.; Yang, H.; Ren, L.-B.; Qi, W.; Li, W.-Y.; Zhang, R.; Xu, J.-H. Triglyceride-Glucose Index Predicts Adverse Cardiovascular Events in Patients with Diabetes and Acute Coronary Syndrome. *Cardiovasc. Diabetol.* **2020**, *19* (1), 80. https://doi.org/10.1186/s12933-020-01054-z.

[63] Avagimyan, A.; Pogosova, N.; Fogacci, F.; Aghajanova, E.; Djndoyan, Z.; Patoulias, D.; Sasso, L. L.; Bernardi, M.; Faggiano, A.; Mohammadifard, N.; Neglia, D.; Carugo, S.; Cicero, A.; Rizzo, M.; Biondi-Zoccai, G.; De Caterina, R.; Sarrafzadegan, N. Triglyceride-Glucose Index (TyG) as a Novel Biomarker in the Era of Cardiometabolic Medicine. *Int. J. Cardiol.* **2025**, *418*, 132663. https://doi.org/10.1016/j.ijcard.2024.132663.

**Q5:** Considering the descriptive characteristics of the AMI group, the authors should reconsider modifying the title and approach to the model as a predictive model for assessing long-term prognosis in patients with preserved ejection fraction.

**A5:**

Thank you for your insightful suggestion. After careful analysis and discussion, we believe that modifying the title to "Predictive Model for Assessing Long-term Prognosis in Patients with Preserved Ejection Fraction" may not be appropriate for the following reasons:

1. In our dataset, 22 AMI patients had an EF < 50%, with 6 of them belonging to the MACE group. Our analysis showed that a reduced ejection fraction was not a significant prognostic factor for AMI patients, as there was no statistical difference in the incidence of reduced EF between groups (this has been supplemented in Table 2).

|  | AMI (n=185) | | |
| --- | --- | --- | --- |
|  | Non-MACE (n=154) | MACE (n=31) | *P* |
| EF＜50%, n (%) | 16（10.39） | 6（19.35） | 0.270 |

(Red words in **Table 2**, page 31, yellow highlight)

2. Among the 185 AMI patients in our study, 22 patients (12%) had an EF <50%, including 6 patients (19%) in the MACE group and 16 patients (10%) in the NO-MACE group. Given that this is a single-center study with a relatively small sample size, further excluding patients with reduced EF would significantly decrease the sample size, potentially compromising the statistical power and reliability of our findings.

For these reasons, we believe that modifying the title in the suggested manner may not accurately reflect the scope and objectives of our study. We appreciate your recommendation and are open to any further suggestions. In our future multicenter study with a larger sample size, we will consider conducting separate analyses for patients with preserved and reduced ejection fraction to further explore their respective prognostic implications.
